# Supplementary figures and images for: SPRY domains encode ubiquitin ligase specificity for ZAP and RIG-I
Source: PLoS Pathog. 2026 May 8;22(5):e1014195. doi: 10.1371/journal.ppat.1014195 (PMC13175477; doi:10.1371/journal.ppat.1014195)

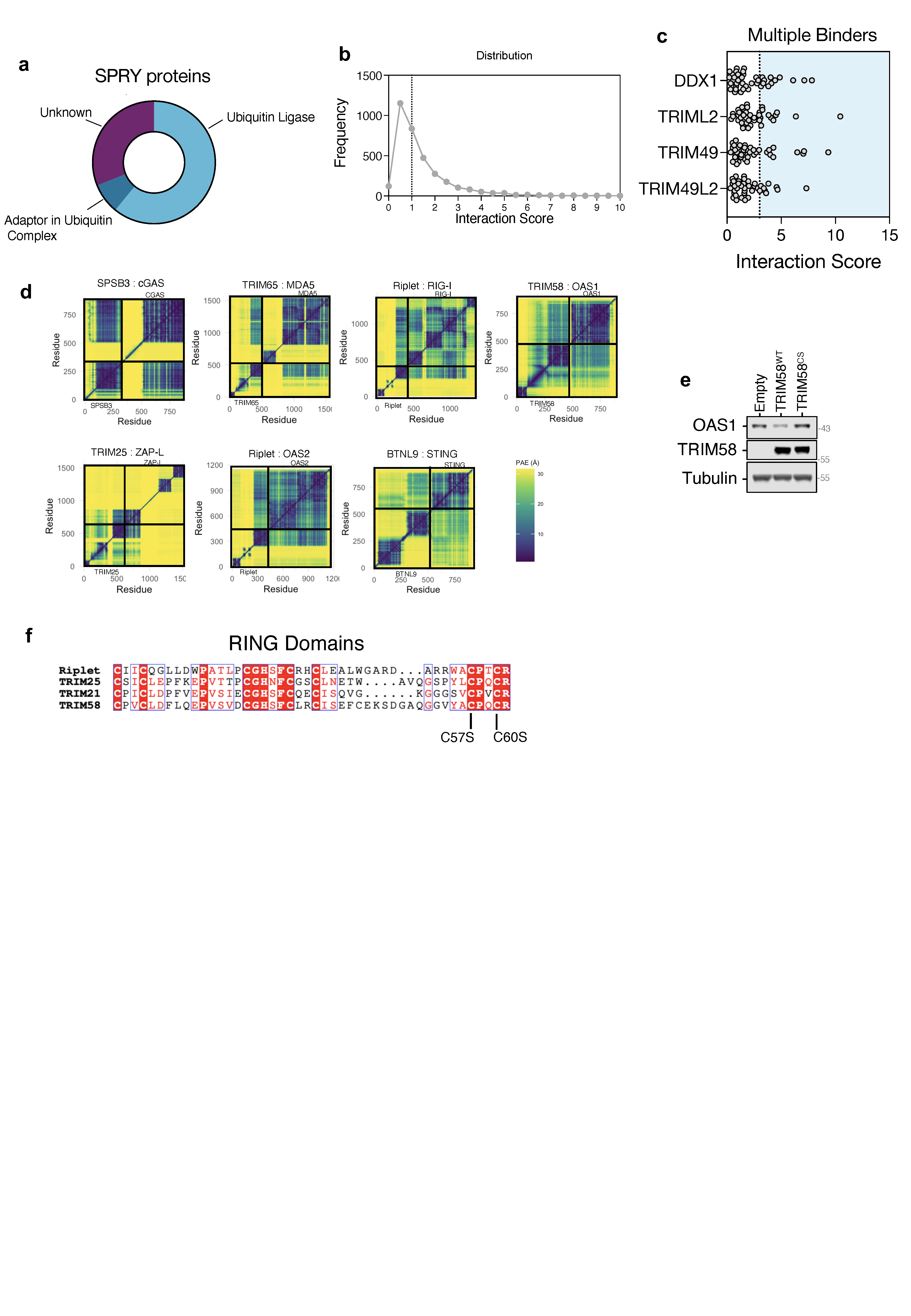

Supplement: S1 Fig — Proportion of known biological functions of all human SPRY-containing proteins (a). Distribution of interaction scores across all SPRY-sensors pairs (b). SPRY-containing proteins with the largest number of interactor partners in the AlphaFold prediction screen (c). PAE maps of selected sensors and SPRY-domain containing proteins (d). HEK293T cells were transfected with plasmids encoding OAS1, wildtype TRIM58 or a catalytically inactive mutant of TRIM58 (TRIM58 C57S C60S). Cells were lysed 48h after transfected and OAS1 levels was measured by western blotting analysis (e). Multiple sequence alignment of the RING domains of Riplet, TRIM25, TRIM21 and TRIM58 (f). (TIFF) [file ppat.1014195.s002.tiff]

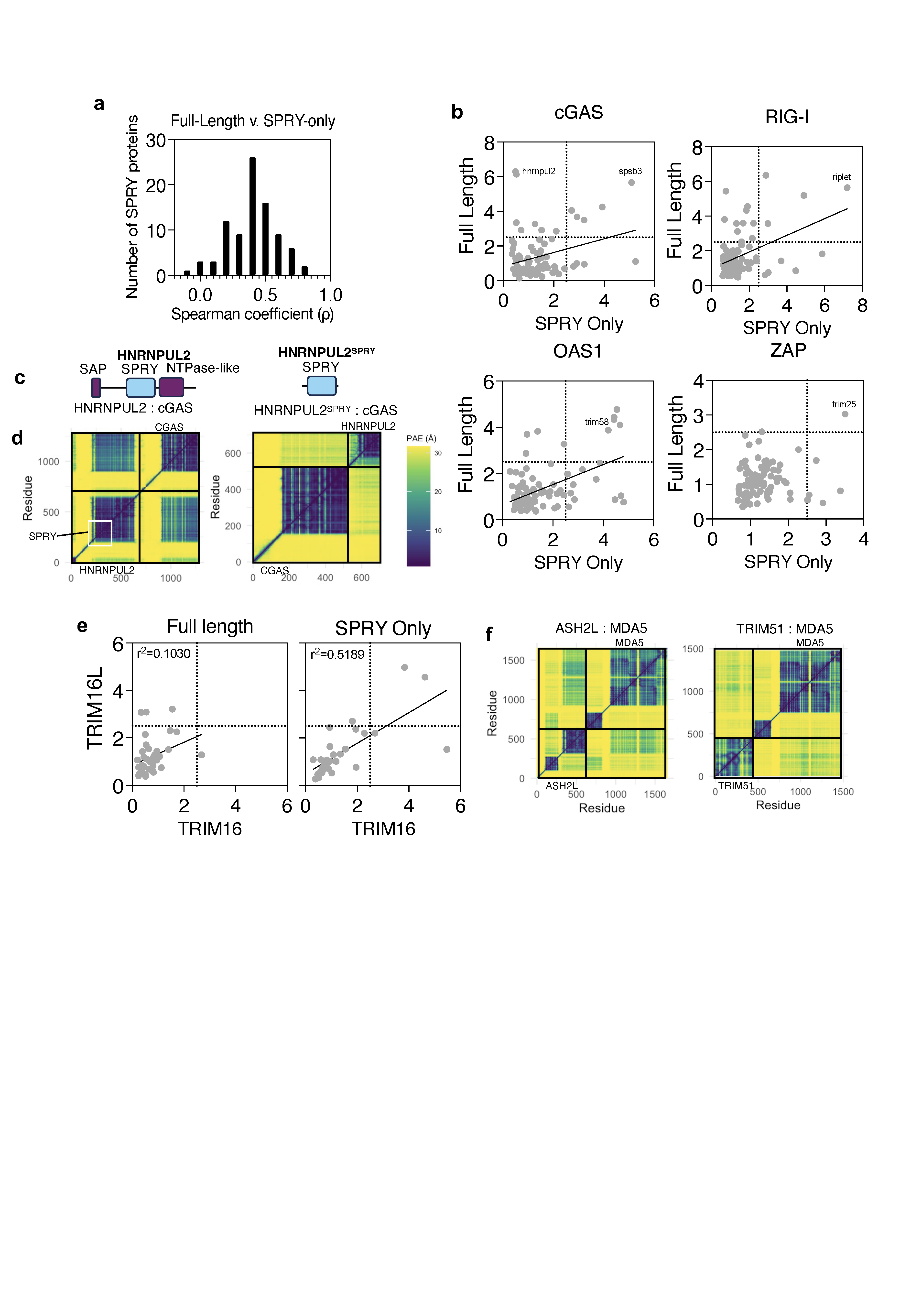

Supplement: S2 Fig — Frequency distribution of Spearman’s correlation coefficients between Full-Length and SPRY-only AlphaFold prediction datasets (a). Comparison of interaction scores obtained from Full-Length and SPRY-only datasets for cGAS, RIG-I, OAS1 and ZAP (b). Schematic represation of the topological organization of hnRNPUL2: an N-terminal DNA-binding SAP domain, a central SPRY domain and a C-terminal AAA/NTPase-like domain (c). PAE maps of hnRNPUL2 full-length and SPRY only interactions with cGAS (d). Comparison of interaction scores obtained from Full-Length and SPRY-only datasets for TRIM16 and TRIM16 (e). PAE maps of ASH2L:MDA5 and TRIM51:MDA5 predicted interactions (f). (TIFF) [file ppat.1014195.s003.tiff]

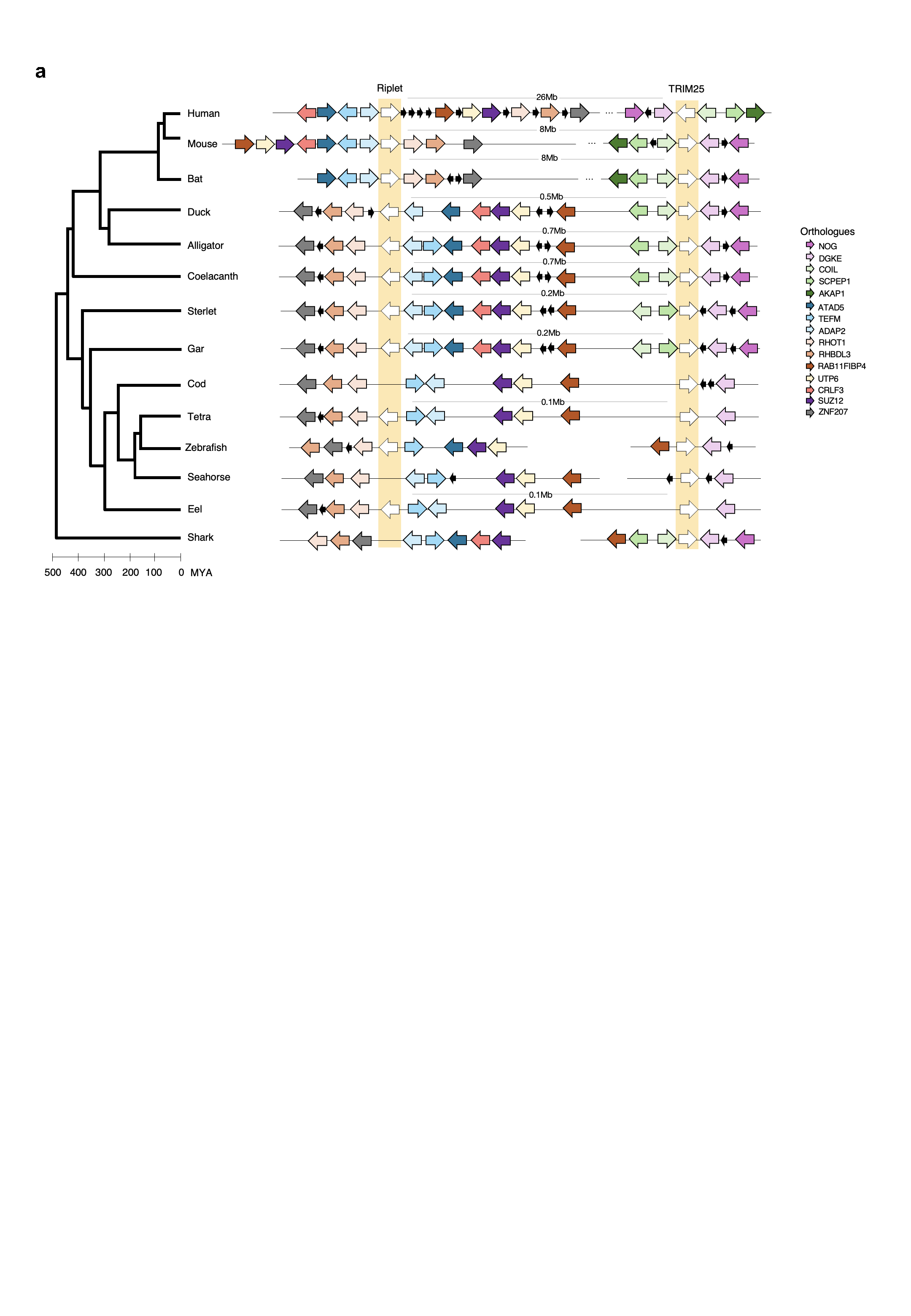

Supplement: S3 Fig — Diagram of the genetic organization of TRIM25 and Riplet loci (in yellow) in vertebrate genomes (a). Mb, megabase (1 million base pairs). MYA, Million years ago. (TIFF) [file ppat.1014195.s004.tiff]

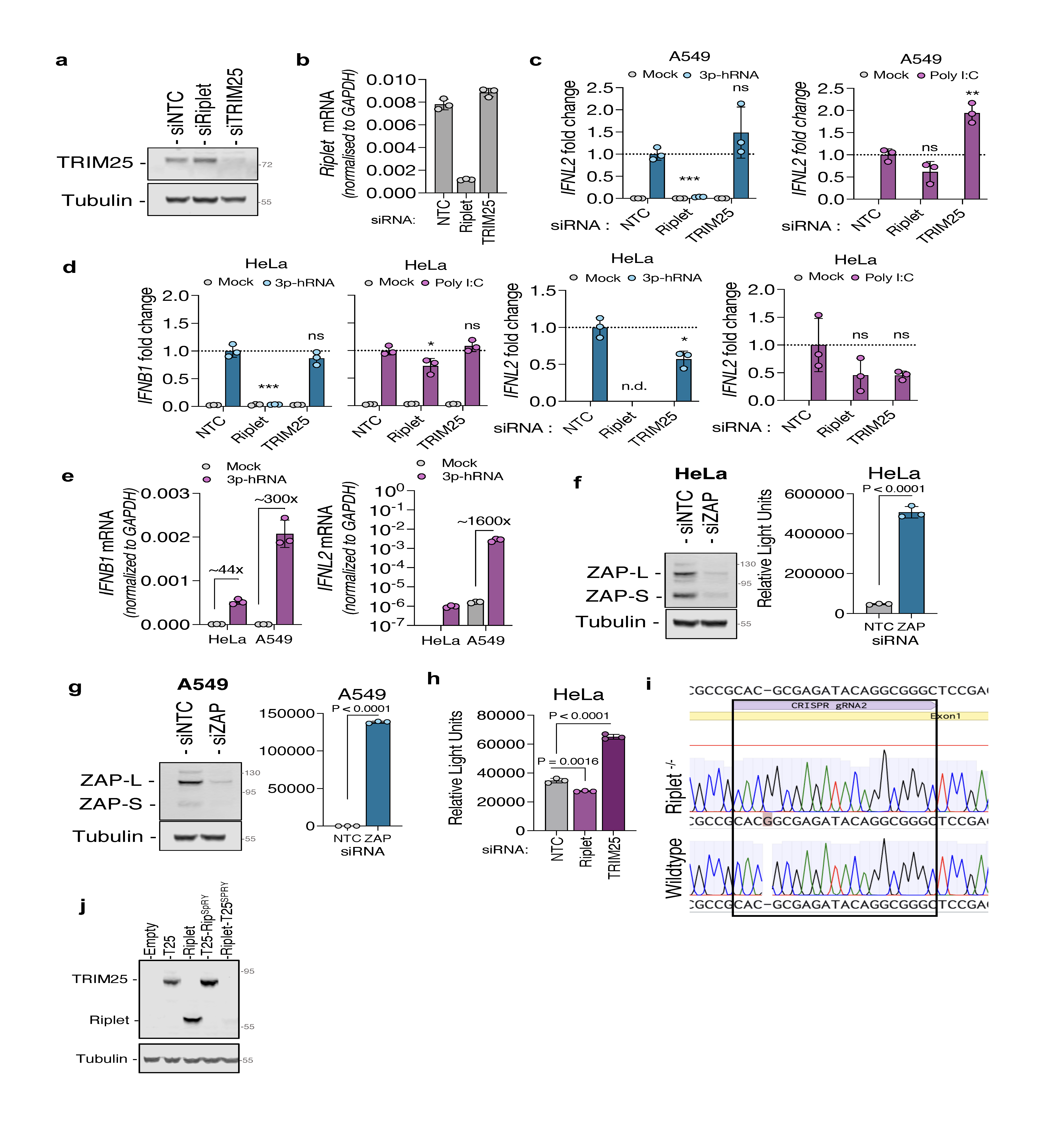

Supplement: S4 Fig — TRIM25 and Riplet were depleted from A549 cells using siRNAs and 48h later cells were analyzed by western blotting (a) and qPCR (b). Riplet and TRIM25 were depleted from A549 cells using siRNAs and 48h later cells were transfected with 3p-hRNA or poly I:C. After 8h after transfections, RNA was extracted and IFNL2 was quantified by qPCR (c). Riplet and TRIM25 were knocked-down from HeLa cells using siRNAs; cells were then transfected with 3p-hRNA or poly I:C and, after 8h, RNA was extracted and IFNB and IFNL2 transcripts were quantified by qPCR (d). IFNB1 and IFNL2 fold-induction in HeLa and A549 cells treated with 3phRNA at 8h post-transfection (e). ZAP was depleted from HeLa cells (f) or A549 cells (g) using siRNAs. After 48h, cells were analyzed by western blotting or infected with a ZAP-sensitive EV71. Luciferase activity was measured 24h later. Riplet- or TRIM25-depleted HeLa cells were infected with ZAP-sensitive nLuc-EV71, and 24h later luciferase activity was quantified (h). Sequencing trace of the RNF135 exon 1 genomic locus in wildtype of Riplet-/- A549 cells (i). A549 Riplet-/- cells were transduced with retroviral vectors encoding the indicated HA-tagged proteins. Protein expression was measured by western blotting (j). (TIFF) [file ppat.1014195.s005.tiff]

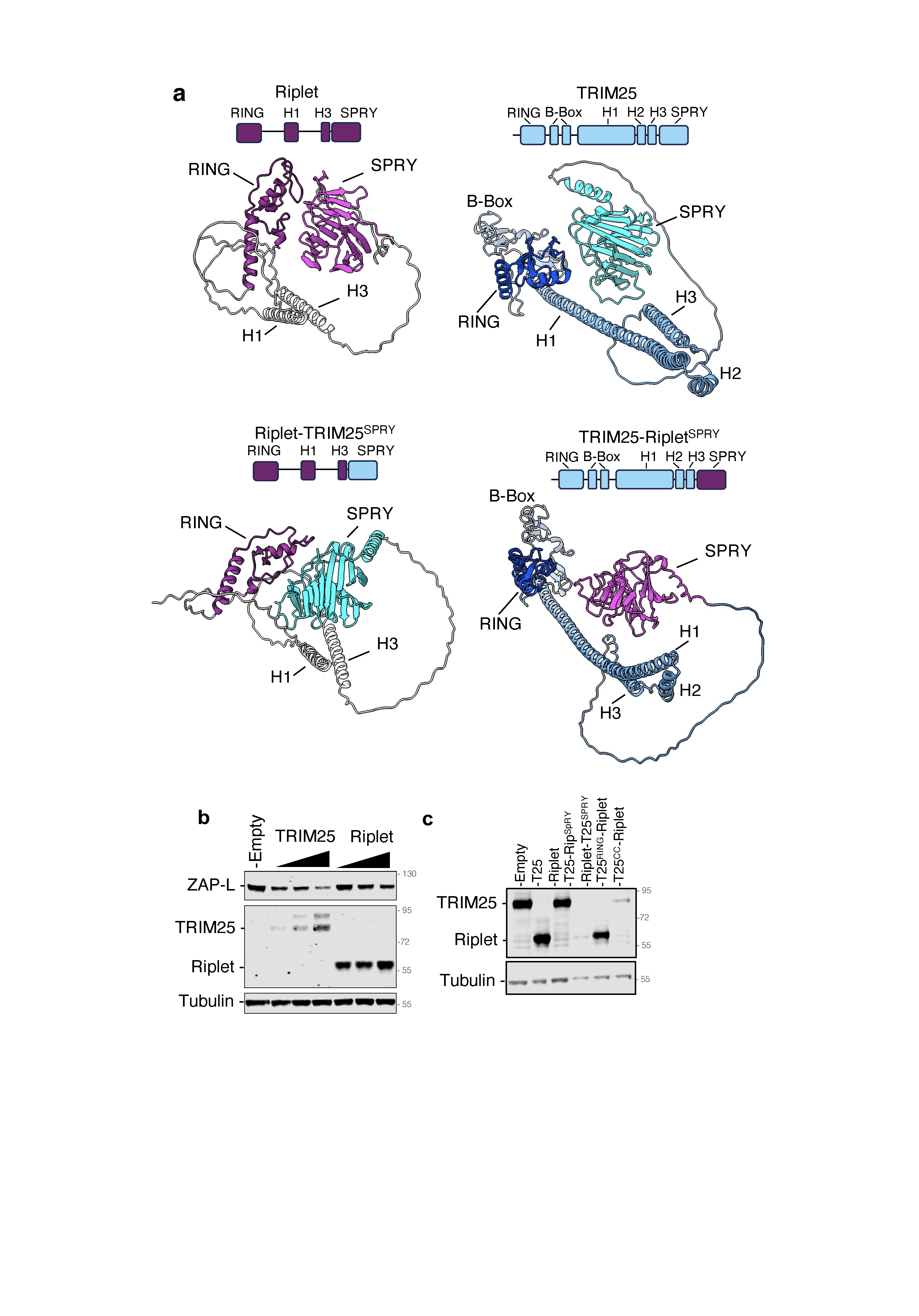

Supplement: S5 Fig — Structural representation of AlphaFold prediction of Riplet, TRIM25 full-length protein and the Riplet-T25SPRY and TRIM25-RipletSPRY chimeric proteins (a) HEK293T TRIM25-/- ZAP-/- were transfected with increasing amounts of plasmids encoding TRIM25-HA or Riplet-HA and ZAP-L expression was analyzed by western blotting (b). A549 Riplet-/- cells were transduced with retroviral vectors encoding the indicated HA-tagged proteins. Protein expression was measured by western blotting (c). (TIFF) [file ppat.1014195.s006.tiff]

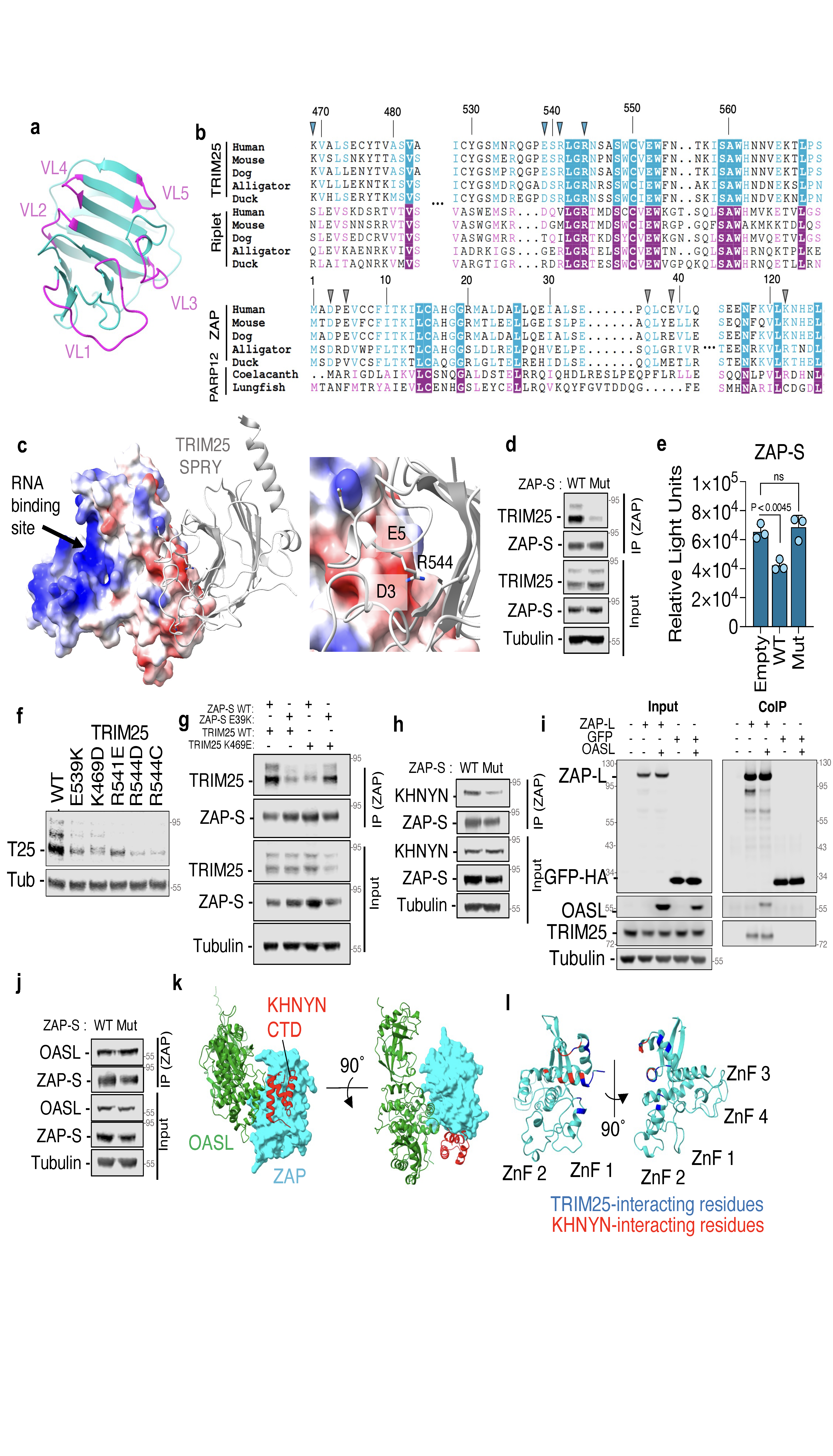

Supplement: S6 Fig — Multiple sequence alignment of orthologues of TRIM25, Riplet, ZAP and PARP12 (b). Predicted structure of ZAP-TRIM25 showing electrostatic surface (c). Inset showed positively charged R544 residue of TRIM25 accommodated by a negatively charged pocket formed by residues D3 and E5 of ZAP (c). HEK293T ZAP-/- TRIM25-/- cells were transfected with plasmid encoding wildtype or qMut ZAP-S along with TRIM25 followed by co-immunoprecipitation of ZAP-S and western blotting (d). Infection of ZAP-/- cells reconstituted with wildtype of qMut ZAP-S (e). Expression levels of single mutants of TRIM25 as indicated (f). HEK293T ZAP-/- TRIM25-/- cells were transfected with plasmid encoding the indicated mutants of ZAP-S and TRIM25 followed by co-immunoprecipitation of ZAP-S and western blotting (g). HEK293T ZAP-/- TRIM25-/- cells were transfected with plasmid encoding wildtype or qMut ZAP-S along with KHNYN followed by co-immunoprecipitation of ZAP-S and western blotting (h). HEK293T were transfected with plasmids encoding ZAP-L-HA, GFP-HA and OASL; cell lysates were used to purified ZAP- or GFP-complexes using an anti-HA antibody and analyzed by western blotting (i). HEK293T ZAP-/- TRIM25-/- cells were transfected with plasmid encoding wildtype or qMut ZAP-S along with OASL followed by co-immunoprecipitation of ZAP-S and western blotting (j). Structure of the RNA-binding domain of ZAP (in blue) and the C-terminal domain of KHNYN (red, KHNYN CTD, obtained from PDB:9BGL) and the predicted sturcture of OASL (in green) (k). Predicted structured of the RNA-binding domain of ZAP (light blue) with residues known to interact with KHNYN in red (described by Bohn et al. [25]) and residues predicted to interact with TRIM25 in blue (l). (TIFF) [file ppat.1014195.s007.tiff]
