# Supplementary material for: SPRY domains encode ubiquitin ligase specificity for ZAP and RIG-I
Source: PLoS Pathog. 2026 May 8;22(5):e1014195. doi: 10.1371/journal.ppat.1014195 (PMC13175477; doi:10.1371/journal.ppat.1014195)
Supplement: Attachment — Submitted filename: 162667_1_rebuttal_3643467_t96bxx.pdf [file ppat.1014195.s008.pdf]

Revision Plan

Review COMMONS

Manuscript number: 2025-03271
Corresponding author(s): Daniel Gonçalves-Carneiro

[The "revision plan" should delineate the revisions that authors intend to carry out in response to the points raised by the referees. It also provides the authors with the opportunity to explain their view of the paper and of the referee reports.

The document is important for the editors of affiliate journals when they make a first decision on the transferred manuscript. It will also be useful to readers of the reprint and help them to obtain a balanced view of the paper.

If you wish to submit a full revision, please use our "Full Revision" template. ***It is important to use the appropriate template to clearly inform the editors of your intentions.****]*

1. General Statements [optional]

We thank reviewers for the general positive feedback and insightful suggestions. Reviewers suggested both editorial changes to improve the narrative of the manuscript and additional experiments to strengthen the conclusions of the study. We agree with both types of suggestions and decided to modify our manuscript accordingly. We started acquiring experimental data to address the reviewers' comments in the following areas:

- Evidence of RIG-I post-translation modifications and sensitivity to proteasomal degradation upon activation
- Evidence of the relevance of TRIM25-interacting residues in the ZAP-S instead of ZAP-L
- Evidence of the effects of TRIM25-Riplet chimera expression in virus infection/RIG-I activation
- Additional bioinformatic analyses, predicted structures and PAE maps

2. Description of the planned revisions

Insert here a point-by-point reply that explains what revisions, additional experimentations and analyses are planned to address the points raised by the referees.

Our replies to each comment are written below in blue font.

Reviewer #1 (Evidence, reproducibility and clarity (Required)):

The authors present a rational, AlphaFold-based strategy to systematically identify interactions between human nucleic acid sensors and SPRY-containing proteins. Their findings demonstrate that SPRY domains encode substrate-

specific recognition patterns that govern immune responses: TRIM25-ZAP in antiviral defense and restricts LNP-encapsulated RNA, while Riplet-RIG-I for the IFNB1 production and restricts lipofection. They further dissect residue-level contributions to the ZAP-TRIM25 interface by integrating structural predictions with experimental validation.

Specific comments.
1. The title of this manuscript appears quite broad given that this study mostly focuses on just TRIM25-ZAP and Riplet-RIG-I pairs.

We agree that the original title was broader than the main mechanistic focus of the study. We will therefore revise the title to better reflect that the manuscript primarily dissects SPRY-domain–mediated specificity in the TRIM25-ZAP and Riplet-RIG-I interactions (identified through our AlphaFold-based screening framework), while retaining the broader screening context. Proposed new title: *"SPRY domains encode ubiquitin ligase specificity for ZAP and RIG-I"*

2. In Figure 1b, several predicted interaction scores appear inconsistent with previously reported experimental interactions. For instance, KHNYN has been experimentally validated as a TRIM25-interacting protein, yet its interaction score is notably low in your computational results. Could the authors clarify whether this discrepancy arises because the TRIM25 SPRY domain does not significantly contribute to KHNYN binding?

We thank the reviewer for raising this point. To our knowledge, published data only support co-immunoprecipitation of TRIM25 and KHNYN in ZAP-deficient in cells (PMID: 31284899), but this does not by itself demonstrate a direct binary interaction, as the association could be mediated by other factors. Consistent with this, our AlphaFold-based screen predicts a low interaction score between KHNYN and TRIM25, suggesting that this may not be a direct protein-protein interaction. Nevertheless, we concede that our approach may have missed interactions that are governed by a small number of interacting residues. We added the following sentences on the limitation of this approach for such interactions in our discussion:

"While our screen revealed novel interactions between SPRY domain containing proteins and innate immune sensors, it is plausible that certain interactions were missed. Interactions that rely on a small number of contacting residues or interactions that may be mediated by a third binding partner are likely to score poorly in our approach. Future optimization of our algorithm will improve the detection of such interactions."

3. In Figure 2c, the authors provide intriguing examples for shared targets by SPRY proteins with quite low homology, and distinct target profiles by nearly identical SPRY domains. However, the underlying mechanisms responsible for these observations are not discussed.

This is an important point. At present, we cannot assign a single definitive mechanism for every example, but there are several plausible explanations consistent with our framework. First, our analysis indicates that substrate recognition is often driven by a limited subset of residues at the interaction surface, such that distinct sequences can converge on similar three-dimensional interface chemistry, while small local differences can shift binding preferences. Second, we note that although a large fraction of predicted contacting residues are within SPRY domains, other domains can also contribute to interaction and substrate recognition, which could modulate binding profiles even when SPRY sequences are near-identical. Third, the Pearson's correlation coefficient was calculated all scores, which may include structures with low confidence scores or low interaction scores <2.5. To address the reviewer's comment, we will repeat this analysis using only structural predictions with high confidence scores and high interaction scores.

4. In Figure 3e and 3f, the authors state that the Riplet-T25 SPRY chimeric protein showed enhanced AlphaFold predicted interaction with ZAP, and validated the interaction experimentally. However, the Alphafold also predicted that an increased interaction for the T25-Riplet chimera, although this mutant failed to be co-precipitated with ZAP. How do the authors reconcile this discrepancy between prediction and experimental outcome?

The reviewer noticed an important nuance in Fig. 3e. AlphaFold predicts that the TRIM25 chimera containing the Riplet SPRY domain (T25–Riplet) has a higher interaction score with ZAP than Riplet alone (Fig. 3e), yet this chimera is not recovered in ZAP co-immunoprecipitation (Fig. 3f). We reconcile this by emphasising that our framework uses an empirically benchmarked threshold: known SPRY–sensor interactions typically score >2.5, and we therefore adopted >2.5 as the cutoff for "high-confidence" candidate interactions. While the T25–Riplet chimera shows an increased score relative to Riplet, its score remains below this >2.5 cutoff in Fig. 3e (which reports interaction scores of the chimeras against ZAP). Therefore, the model is consistent with the experimental outcome: AlphaFold suggests some degree of interface compatibility, but not at a level we would classify as a robust/predictive interaction under our validated threshold. We clarified this point in the Results section to explicitly note that sub-threshold "increases" should be interpreted cautiously:

"Using the T25-RipletSPRY instead of the Riplet protein, predicted a higher interaction score despite the lack of specific pull-down between this chimera and ZAP; importantly, this interaction score is below our defined threshold (2.5), highlighting the importance of benchmarking predicted scores against known interactions."

5. It is curious if the authors explain why TRIM25 consistently appears as two bands in many of the presented figures.

We have also pondered this observation. Other studies report the double band pattern in western blots of TRIM25 (PMID: 17392790, 28060952, 21292167) and it is believed

to be a product of non-degradative self-ubiquitination of TRIM25, primarily acting on the K117 residue (PMID: 21292167). We will add a brief description of this phenomenon in the figure legend.

6. In Figure 4b, the authors show that treatment with a proteasome inhibitor increased RIG-I ligand-induced IFNB1 expression and propose that RIG-I may undergo rapid degradation following its interaction with Riplet. However, the evidence supporting this claim is weak. The authors should demonstrate: (1) that RIG-I is indeed degraded via the proteasome, and (2) whether RIG-I undergoes K48-linked ubiquitination. Mutational analysis of putative ubiquitination sites in RIG-I would help clarify its contribution to the observed IFN responses.

This is an important point and we are currently performing experiments addressing these questions. Specifically we will provide evidence of (1) whether RIG-I is degraded after activation using a combination of western blotting and pharmacological inhibition of the proteosome/translation machinery; (2) whether RIG-I goes K48- or K63-mediated ubiquitination by performing coIPs of RIG-I in the presence of HA-Ub wildtype or the commonly used HA-Ub K48 and K63 mutants (PMID: 15728840); and (3) whether lysine-to-arginine substitution of key residues impacts RIG-I ubiquitination/degradation.

7. Figure 5 c-g: why do the authors show ZAP-L, but not ZAP-S?

Both ZAP-S and ZAP-L isoforms contain identical N-terminal domains, which is the region that interacts with TRIM25. Therefore, we assumed that the interaction between TRIM25 and ZAP-L would be similar to TRIM25-ZAP-S. However, to test this assumption, we will generate equivalent mutations in ZAP-S and perform similar co-immunoprecipitation experiments.

Reviewer #1 (Significance (Required)):

This manuscript starts with the AlphaFold-based screening of interactions between human nucleic acid sensors and SPRY-containing proteins. However, the authors then just focused on TRIM25-ZAP and Riplet-RIG-I, whose interactions have been well demonstrated previously, although other protein interactions were not further explored. Also, the information on the evolutional aspects of TRIM25, ZAP, Riplet and RIG-I did not lead to clear conclusions. The differential contribution of TRIM25-ZAP and Riplet-RIG-I in LNP- and lipofectamine-transduced RNAs is interesting, although data shown in Fig.6 are expected from previous studies, and are not so relevant to other data in this study.
Therefore, the study is not well integrated, although pieces are interesting.
This study may attract researchers in innate

My expertise is innate immunity and RNA biology.

Reviewer #2 (Evidence, reproducibility and clarity (Required)):

The paper describes the discovery of unknown E3-RNA sensor interactions from a large scale in silico prediction screen, as well as the clarification of previously described E3-sensor interactions. These findings extend previous work showing ancient relationships between nucleic acid sensors and RING E3s (e.g. PMID: 33373584), which also described the RIPLET-RIG-I pairing identified in the present screen.

The interactions focused on are shown to have functional implications for immune signaling pathways, and stability implications for the bound sensor. The argument for the screen is that E3-target interactions are often too transient to detect biochemically. While possibly true, several of the pairings are confirmed by co-IP, with either WT E3 or a catalytically deficient E3 (known elsewhere as a 'substrate trap').

The key conclusions are convincing and interesting; in particular, the conserved interactions between RIPLET and RIG-I, and TRIM25 and ZAP. The hypothesis that the two E3s arose from a common ancestor is intriguing, and the use of chimeras in functional experiments suggest that the length of the coiled coil domains contributes to substrate targeting. The most interesting observation IMO is that showing that RNA vaccines can be sensed by orthogonal sensor/E3 pathways, depending on transfection method, suggesting that distinct entry routes are surveyed by different sensors. These experiments are well performed as E3 manipulation phenocopies sensor manipulation, supporting that the in silico approach will ID relevant pairings.

Including the PAE plots for some of the key interactions would be helpful, as a lot of the interaction confidence metrics are hidden in interaction 'scores'. Fig. 1b heatmap is presented as a row max, so it is difficult to calibrate one E3 against another. The raw data from e.g. fig. 1c would be a valuable addition. This would also help orientate future studies predicting similar protein-protein interactions.

We agree with the reviewer and we will provide the raw values for the interaction scores and PAE maps as supplementary data to be included in the final publication.

Figure 1 appears to just use the isolated SPRY domain for screening - were full-length proteins used?

The data in Figure 1 was generated using full-length proteins, but it is interesting to see if a similar screen with SPRY domains alone can replicate the predicted interactions. We will repeat this using SPRY domain sequences.

How many copies of the FL protein were used. TRIM5 employs a low affinity, high avidity binding method; do binding patterns change when the valency of either component is altered? The Alphafold approach perhaps selects for high affinity

Review COMMONS

binders? I don't expect many more experiments to be done here, but commenting on this would be useful.

This is a rational consideration that we overlooked. We included in our discussion a comment on the limitation of this approach in the context of multimeric assemblies: *"Similarly, the oligomeric nature of some SPRY-containing proteins [22] is likely to impact the correct placement of these domains and, therefore, impact the predicted interaction score. Future optimization of our algorithm will improve the detection of such interactions."*

The TRIM25 -Riplet PRYSPRY swap experiments in Figure 3 are very informative and powerful. Some more detail on domain boundaries used would be helpful, including AF predictions of what these chimeras look like with respect to their natural counterparts.

We agree with the reviewer about the need to explicit define domain boundaries. We will include as supplemental information a comparison of the AF prediction of these chimeras in relation to the native proteins.

While AF can place confidence metrics on domain-domain interactions, SPRY containing proteins are themselves often comprised of regions of high structural confidence (e.g. many available PDBs for RINGs, coils and SPRYs) but their relative arrangement within the molecule is unpredictable. Do isolated SPRYs show any better/worse binding to targets?

This is a good point as well, and this can be addressed by repeating the AlphaFold screen using only SPRY domain proteins rather than full-length protein (as described above).

Technically, fig. 1f does not show that TRIM58 destabilises OAS1, as there is no condition with OAS1 alone. Perhaps alter the text to reflect this or repeat with the necessary control. The direction of the text is fine, as Fig. 1g provides a striking result, but 1f needs attention.

The reviewer raises an important consideration. To address this, we will repeat the experiment using a OAS1 alone condition, as suggested.

Fig. 2c - for clarity, please specify the meaning of the connecting lines between the bait 'hits' in the legend. What does the correlation coefficient relate to exactly? % similarity, is this across the whole molecule, or the PRYSPRY (presumably the latter would be a more useful comparison). And it is well established that single variations in SPRY variable loops can toggle binding, so this could be better referenced in the text. It would also be helpful to see e.g. dissimilar PRYSPRYs binding a common target, as PAE plots in the supplementary. Do any shared motifs occur at the variable loops between dissimilar SPRY molecules?

Review COMMONS

We agree that this figure needs more clarity. The connecting lines in Fig. 2c indicate protein-protein predictions with common sensors, i.e. connecting lines between the interaction score of ASH2L-MDA5 and the interaction score of TRIM51-MDA5. We only use % similarity of the SPRY domain alone, not the whole molecule.
We have modified the figure legend to clarify this point and we include the PAE maps as supplementary information, as requested.

Fig. 2i - Bat RIG-I binds both TRIM25 and Riplet? This is in contrast to the predicted directionality in 2h?

The reviewer astutely noted that, in Fig.2i, pulling down bat RIG-I co-immunoprecipitated with both bat Riplet and bat TRIM25, while AlphaFold predictions only suggest a Riplet-RIG-I interaction. However, while bat Riplet and bat TRIM25 express at comparable levels in the input sample, bat Riplet was far more enriched in RIG-I pulldowns than bat TRIM25. Our interpretation of this data is that, indeed, bat Riplet-RIG-I interaction is more powerful than TRIM25-Rig-I.

Fig. 3a-b, Sup Fig. 3c,d - IFNB1 transcript normalised to 3p-hRNA transfection in control NTC cells - the presentation chosen obscures the baseline IFNB1 levels in the different siRNA transfections. What is the fold induction of IFNB1 in the different cell lines?

We will include the fold induction in each cell line as supplementary information.

Fig. 3g - RLUs of EV-A71 is only tested in TRIM25 KO cells transfected with the Riplet T25 chimera. The full panel of cDNAs (parental E3s and the inverse 25-riplet swap) should be tested in parallel to confirm the effect is specific to TRIM25 PRYSPRY.

This is a great suggestion that will help clarify the role of different domains of TRIM25 in its antiviral activity. We are currently generating cell-line stably expressing these truncations and will perform the suggested experiment.

Fig. 4b - time point of 3p-hRNA transfection? Y-axis label suggested normalisation to NTC - incorrect label? What is the effect of bortezomib on IFNB1 mRNA in mock treated cells?

We thank the reviewer for spotting this typo, we have known corrected the axis label. We harvested cellular mRNA 8h post-transfection. Bortezomib-treatment slightly reduced the background expression of IFNB1 mRNA, but this signal is very close to the detection limit that it is difficult to draw conclusions. Nevertheless, the addition of bortezomib did not increase IFNB1 mRNA expression in the absence of a stimulus.

Fig. 4g - these experiments would benefit from an untransfected control cell to clarify how cDNA expression impacts sensor stability.

Revision Plan

We agree with the reviewer and we will include an untransfected control.

There seems to be an inverse correlation between sensor degradation and signaling output - is that the summary of Fig. 4? On the one hand, sensor degradation attenuates functional output (Fig. 4b), and the E3 that degrades the sensor is required for sensor function; on the other hand, changing coil-length in the E3 disables sensor degradation (Fig. 4g) but and enhances signaling response (Fig. 3j). Do the chimeras of panel Fig. g, h influence IFNB1 expression in the assay from Fig. 3j - this experiment would marry RIG-I expression with signal output.

This is an interesting experiment. We are in the process of generating a TRIM25$^{-/-}$ Riplet$^{-/-}$ cell line, which we will use to reconstitute with the chimeras mentioned above and perform the requested experiment.

The data is generally clear. To facilitate their interpretation and for clarity, Western blots require size markers and Co-IPs should indicate the flag-/ha-epitope tags. Would make fig. 2 i-j much clearer, particularly given apparent co-migration of IgG (heavy chain?) and riplet, and the lack of control IPs.

We agree that contextual markings will improve the interpretation of these results. We will add size markers to the western blots in fig2 in order to improve clarity.

The figure legends could provide more detail.

We will add additional experimental details (such as time points) to the figure legends.

Reviewer #2 (Significance (Required)):

The paper provides a rich resource of potential E3-sensor interactions and represents a conceptual and technical advance for the field. The authors take a novel approach to identify these pairings. Several pairings are validated in CoIPs,and two pairings (T25-ZAP, RIPLET-RIG-I) are studied in detail. Many E3s - including the TRIM proteins which comprise the bulk of E3s studied here - are purported to regulate key nucleic acid sensors in the literature, but the large scale approach taken here provides evidence that the pairings are really quite specific. The findings also supports prior work showing that the PRYSPRY domain (here called the SPRY) is a functionally plastic module that through variable loops can bind a range of different protein substrates.

The paper will be most interesting to the innate immune field, those working on nucleic acid sensing, and those looking at innate responses to RNA vaccines.

Regulation of E3 ubiquitin ligases, viral RNA sensing

Revision Plan

Review COMMONS

3. Description of the revisions that have already been incorporated in the transferred manuscript

Please insert a point-by-point reply describing the revisions that were already carried out and included in the transferred manuscript. If no revisions have been carried out yet, please leave this section empty.

We have already included a few changes to the current manuscript including:
- We have included addition comments to the discussion and the results sections that cover points raised by the reviewers (e.g. limitations of the approach, interpretation of interaction scores);
- Added additional details to the figure legends

4. Description of analyses that authors prefer not to carry out

Please include a point-by-point response explaining why some of the requested data or additional analyses might not be necessary or cannot be provided within the scope of a revision. This can be due to time or resource limitations or in case of disagreement about the necessity of such additional data given the scope of the study. Please leave empty if not applicable.
