# Supplementary material for: SPRY domains encode ubiquitin ligase specificity for ZAP and RIG-I
Source: PLoS Pathog. 2026 May 8;22(5):e1014195. doi: 10.1371/journal.ppat.1014195 (PMC13175477; doi:10.1371/journal.ppat.1014195)
Supplement: Attachment — Submitted filename: Response to Reviewers Comments.pdf [file ppat.1014195.s009.pdf]

Reviewer #1 (Evidence, reproducibility and clarity (Required)):

The authors present a rational, AlphaFold-based strategy to systematically identify interactions between human nucleic acid sensors and SPRY-containing proteins. Their findings demonstrate that SPRY domains encode substrate-specific recognition patterns that govern immune responses: TRIM25-ZAP in antiviral defense and restricts LNP-encapsulated RNA, while Riplet-RIG-I for the IFNB1 production and restricts lipofection. They further dissect residue-level contributions to the ZAP-TRIM25 interface by integrating structural predictions with experimental validation.

Specific comments.

1. The title of this manuscript appears quite broad given that this study mostly focuses on just TRIM25-ZAP and Riplet-RIG-I pairs.

We agree that the original title was broader than the main mechanistic focus of the study. We therefore revised the title to better reflect that the manuscript primarily dissects SPRY-domain–mediated specificity in the TRIM25–ZAP and Riplet–RIG-I interactions (identified through our AlphaFold-based screening framework), while retaining the broader screening context. Proposed new title:

"SPRY domains encode ubiquitin ligase specificity for ZAP and RIG-I"

2. In Figure 1b, several predicted interaction scores appear inconsistent with previously reported experimental interactions. For instance, KHNYN has been experimentally validated as a TRIM25-interacting protein, yet its interaction score is notably low in your computational results. Could the authors clarify whether this discrepancy arises because the TRIM25 SPRY domain does not significantly contribute to KHNYN binding?

We thank the reviewer for raising this point. To our knowledge, published data only support co-immunoprecipitation of TRIM25 and KHNYN in ZAP-deficient in cells (PMID: 31284899), but this does not by itself demonstrate a direct binary interaction, as the association could be mediated by other factors. Consistent with this, our AlphaFold-based screen predicts a low interaction score between KHNYN and TRIM25, suggesting that this may not be a direct protein-protein interaction. Nevertheless, we concede that our approach may have missed interactions that are governed by a small number of interacting residues. We added the following sentences on the limitation of this approach for such interactions in our discussion:

“While our screen revealed novel interactions between SPRY domain containing proteins and innate immune sensors, it is plausible that certain interactions were missed. Interactions that rely on a small number of contacting residues or interactions that may be mediated by a third binding partner are likely to score poorly in our approach. Future optimization of our algorithm will improve the detection of such interactions.”

3. In Figure 2c, the authors provide intriguing examples for shared targets by SPRY proteins with quite low homology, and distinct target profiles by nearly identical SPRY domains. However, the underlying mechanisms responsible for these observations are not discussed.

This is an important point. We agree that the mechanistic basis for these observations should be discussed more explicitly. To address this, we compared SPRY sequence similarity with similarity in predicted sensor-binding profiles using both isolated SPRY domains and full-length protein sequences. Across all pairwise comparisons, overall SPRY sequence identity was only weakly associated with binding-profile similarity in both analyses (**FigYa**). Thus, our data suggest that shared targeting is not determined primarily by global SPRY homology. Instead, the data support a model in which substrate recognition depends on a limited set of local interface determinants, such as residues within variable surface loops or additional contacts contributed by regions outside the SPRY domain, that may be conserved across otherwise divergent SPRY proteins. Conversely, closely related SPRY domains may display distinct target profiles if small local sequence differences, or context provided by non-SPRY regions of the full-length protein, alter the physicochemical properties of the interaction surface. We have added text to the Discussion to clarify these points.

FigY

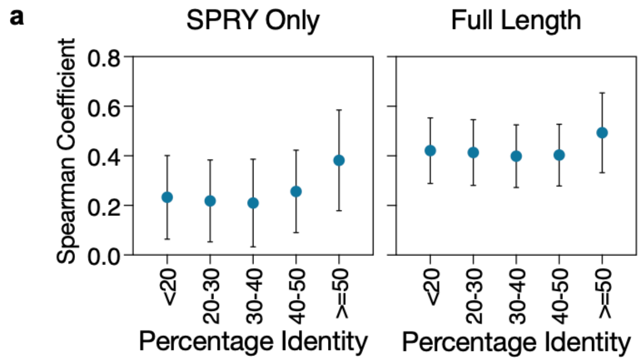

4. In Figure 3e and 3f, the authors state that the Riplet-T25 SPRY chimeric protein showed enhanced AlphaFold predicted interaction with ZAP, and validated the interaction experimentally. However, the Alphafold also predicted that an increased interaction for the T25-Riplet chimera, although this mutant failed to be co-precipitated with ZAP. How do the authors reconcile this discrepancy between prediction and experimental outcome?

The reviewer noticed an important nuance in **Fig. 3e**. AlphaFold predicts that the TRIM25 chimera containing the Riplet SPRY domain (T25–Riplet) has a higher interaction score with ZAP than Riplet alone (**Fig. 3e**), yet this chimera is not recovered in ZAP co-immunoprecipitation (**Fig. 3f**). We reconcile this by emphasising

that our framework uses an empirically benchmarked threshold: known SPRY–sensor interactions typically score >2.5, and we therefore adopted >2.5 as the cutoff for "high-confidence" candidate interactions. While the T25–Riplet chimera shows an increased score relative to Riplet, its score remains below this 2.5 cutoff in Fig. 3e (which reports interaction scores of the chimeras against ZAP). Therefore, the model is consistent with the experimental outcome: AlphaFold suggests some degree of interface compatibility, but not at a level we would classify as a robust/predictive interaction under our validated threshold. We clarified this point in the Results section to explicitly note that sub-threshold "increases" should be interpreted cautiously:

"Using the T25-RipletSPRY instead of the Riplet protein, predicted a higher interaction score despite the lack of specific pull-down between this chimera and ZAP; importantly, this interaction score is below our defined threshold (2.5), highlighting the importance of benchmarking predicted scores against known interactions."

5. It is curious if the authors explain why TRIM25 consistently appears as two bands in many of the presented figures.

We have also pondered this observation. Other studies reported the double band pattern in western blots of TRIM25 (PMID: 17392790, 28060952, 21292167) and it is believed to be a product of non-degradative self-ubiquitination of TRIM25, primarily acting on the K117 residue (PMID: 21292167). We added a brief description of this phenomenon in the figure legend.

6. In Figure 4b, the authors show that treatment with a proteasome inhibitor increased RIG-I ligand-induced IFNB1 expression and propose that RIG-I may undergo rapid degradation following its interaction with Riplet. However, the evidence supporting this claim is weak. The authors should demonstrate: (1) that RIG-I is indeed degraded via the proteasome, and (2) whether RIG-I undergoes K48-linked ubiquitination. Mutational analysis of putative ubiquitination sites in RIG-I would help clarify its contribution to the observed IFN responses.

This is an important point and we performed experiments addressing these questions. We first addressed if RIG-I is indeed degraded by the proteasome using two commonly used proteasome inhibitors, MG132 and bortezomib which both inhibit the activity of the 26S proteasome complex. While we had previously found that treating cells with bortezomib boosted IFNB production after 8h of RIG-I activation, treating cells with MG132 slightly reduced IFNB expression (**FigXa**). Western blot analysis revealed that RIG-I is not degraded within the first 8h post-stimulation with 3phRNA (**FigXb**). Based on this new evidence, we concluded that that the effects of bortezomib on IFNB expression must be due to the stabilization of other components of the RIG-I signalling pathway and not RIG-I itself. However, we

observed that in absent of stimulation, Riplet-depleted cells showed increased levels of endogenous RIG-I, suggesting that Riplet regulates turnover of RIG-I at least in unstimulated cells (**FigXc**). Following the reviewer's suggestion, we searched the scientific literature to identify residues in RIG-I that are ubiquitinated by Riplet and found four reported sites: K164 and K172, located the CARD2 domain, and K849 and K851, located in the C-terminal domain (PMID: 21147464, 27387525). To test if these residues control the Riplet-dependent degradation of RIG-I, we generated two double mutants of RIG-I: (1) K164R K172R RIG-I and (2) K849R K851R RIG-I (**FigXd**). We then co-transfected HEK293T RIG-I$^{-/-}$ cells with wildtype or each double mutant of RIG-I along with Riplet or an empty plasmid (**FigXe**). We found that both mutants of RIG-I were still degraded in the presence of Riplet, suggesting that Riplet-mediated degradation does not involve the indicated residues. Together, these new data suggest that RIG-I residues ubiquitinated by Riplet in resting and stimulated conditions must be different. While we could not identify residues that mediate RIG-I degradation, future investigation using mass spectrometry will identify residues in RIG-I that are modified by Riplet and are involved in its ubiquitination. We have modified **Figure 4** and **Supplementary Fig. 5** to accommodate this.

FigX

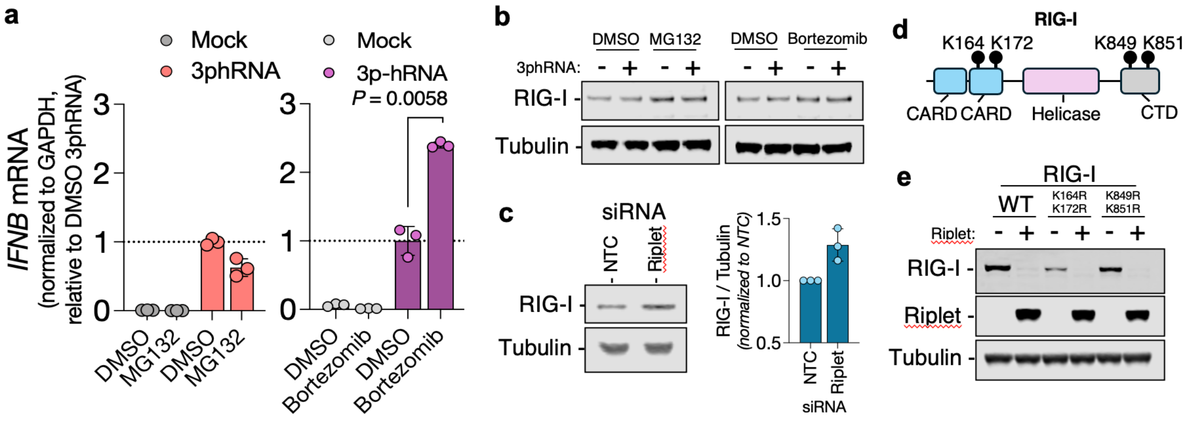

7. Figure 5 c-g: why do the authors show ZAP-L, but not ZAP-S?

Both ZAP-S and ZAP-L isoforms contain identical N-terminal domains, which is the region that interacts with TRIM25. Therefore, we assumed that the interaction between TRIM25 and ZAP-L would be similar to TRIM25-ZAP-S. However, to test this assumption, we generated equivalent mutations in ZAP-S (ZAP-S^{qMut}: D3R, E5R, E39R and K122E) and perform similar co-immunoprecipitation and infection experiments. We found that the interaction between TRIM25 and ZAP-S^{qMut} is almost completely abolished (**FigS56d**) and ZAP-S antiviral activity is also significantly reduced (**FigS6e**). Such interaction could be restored by inverted-charge mutants in TRIM25 (**FigS6g**). Similar to what we observed with ZAP-L, the interaction between ZAP-S and KHNYN was substantially reduced (**FigS6h**), while OASL-ZAP-S interaction was unaffected by these mutations (**FigS6j**). These data support the

conclusion that both ZAP-S and ZAP-L share a similar interface with TRIM25, which is mediated by the indicated residues. These new experiments are now included in Supplementary Figure 6.

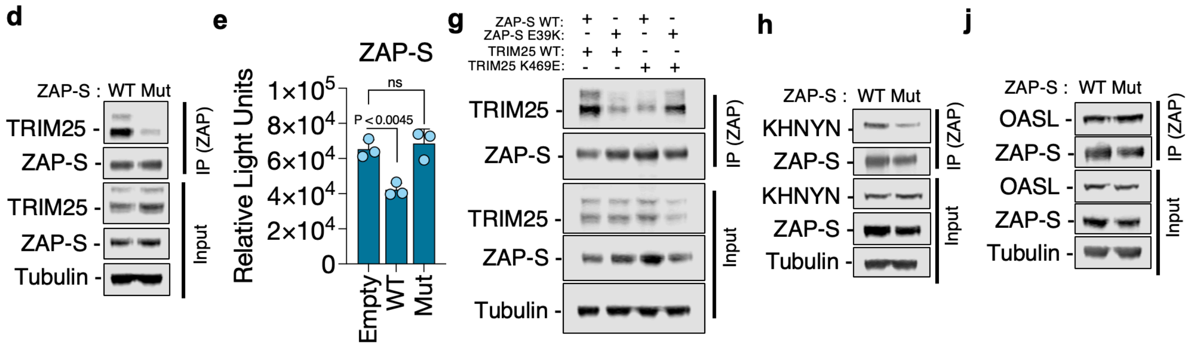

Reviewer #1 (Significance (Required)):

This manuscript starts with the AlphaFold-based screening of interactions between human nucleic acid sensors and SPRY-containing proteins. However, the authors then just focused on TRIM25-ZAP and Riplet-RIG-I, whose interactions have been well demonstrated previously, although other protein interactions were not further explored. Also, the information on the evolutional aspects of TRIM25, ZAP, Riplet and RIG-I did not lead to clear conclusions. The differential contribution of TRIM25-ZAP and Riplet-RIG-I in LNP- and lipofectamine-transduced RNAs is interesting, although data shown in Fig.6 are expected from previous studies, and are not so relevant to other data in this study.
Therefore, the study is not well integrated, although pieces are interesting.
This study may attract researchers in innate

My expertise is innate immunity and RNA biology.

Reviewer #2 (Evidence, reproducibility and clarity (Required)):

The paper describes the discovery of unknown E3-RNA sensor interactions from a large scale in silico prediction screen, as well as the clarification of previously described E3-sensor interactions. These findings extend previous work showing ancient relationships between nucleic acid sensors and RING E3s (e.g. PMID: 33373584), which also described the RIPLET-RIG-I pairing identified in the present screen.

The interactions focused on are shown to have functional implications for immune signaling pathways, and stability implications for the bound sensor. The argument for

the screen is that E3-target interactions are often too transient to detect biochemically. While possibly true, several of the pairings are confirmed by co-IP, with either WT E3 or a catalytically deficient E3 (known elsewhere as a 'substrate trap').

The key conclusions are convincing and interesting; in particular, the conserved interactions between RIPLET and RIG-I, and TRIM25 and ZAP. The hypothesis that the two E3s arose from a common ancestor is intriguing, and the use of chimeras in functional experiments suggest that the length of the coiled coil domains contributes to substrate targeting. The most interesting observation IMO is that showing that RNA vaccines can be sensed by orthogonal sensor/E3 pathways, depending on transfection method, suggesting that distinct entry routes are surveyed by different sensors. These experiments are well performed as E3 manipulation phenocopies sensor manipulation, supporting that the in silico approach will ID relevant pairings.

8. Including the PAE plots for some of the key interactions would be helpful, as a lot of the interaction confidence metrics are hidden in interaction 'scores'. Fig. 1b heatmap is presented as a row max, so it is difficult to calibrate one E3 against another. The raw data from e.g. fig. 1c would be a valuable addition. This would also help orientate future studies predicting similar protein-protein interactions.

We agree with the reviewer and we will provide the raw values for the interaction scores as part of PLoS Pathogens' policy on data submission. We also agree that PAE maps for key interactions would be helpful, so we have included them in **Supplementary Figure 1**.

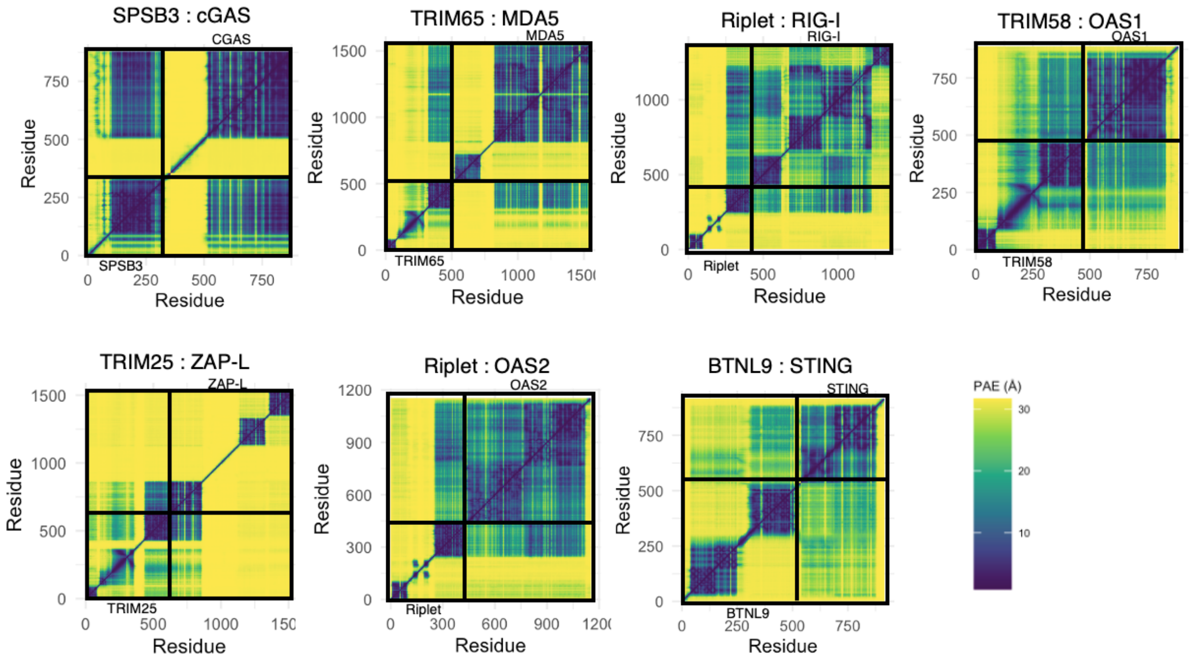

9. Figure 1 appears to just use the isolated SPRY domain for screening - were full-length proteins used?

The data in Figure 1 was generated using full-length proteins, but we have repeated the screen using SPRY domains alone. We included a comparison of these two datasets in **Figure 2** and **Supplementary Figure 2**.

10. How many copies of the FL protein were used. TRIM5 employs a low affinity, high avidity binding method; do binding patterns change when the valency of either component is altered? The Alphafold approach perhaps selects for high affinity binders? I don't expect many more experiments to be done here, but commenting on this would be useful.

This is a rational consideration that we overlooked. We included in our discussion a comment on the limitation of this approach in the context of multimeric assemblies:

"Similarly, the oligomeric nature of some SPRY-containing proteins [22] is likely to impact the correct placement of these domains and, therefore, impact the predicted interaction score. Future optimization of our algorithm will improve the detection of such interactions."

11. The TRIM25 -Riplet PRYSPRY swap experiments in Figure 3 are very informative and powerful. Some more detail on domain boundaries used would be helpful, including AF predictions of what these chimeras look like with respect to their natural counterparts.

We agree with the reviewer about the need to explicitly define domain boundaries. We included boundary detailed in the methods section and, as supplemental information, we included a comparison of the AF prediction of these chimeras in relation to the native proteins (**FigS5a**).

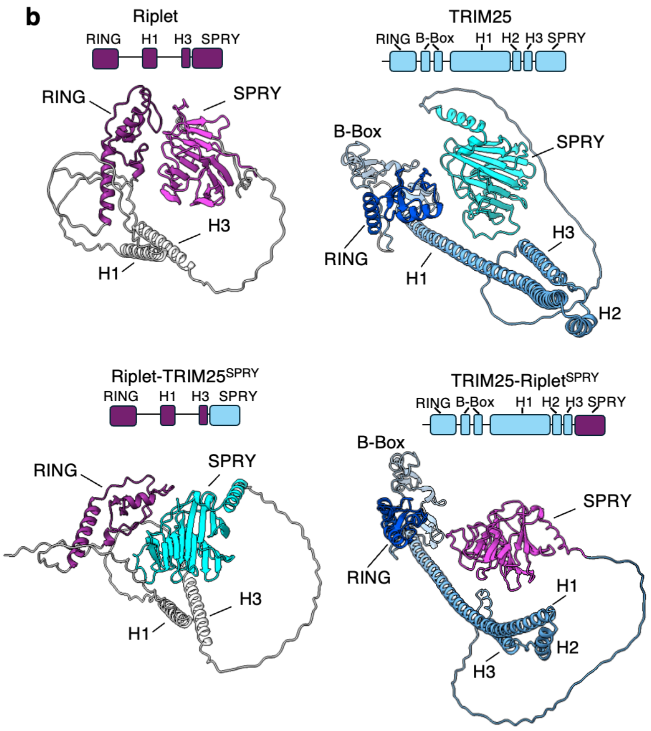

12. While AF can place confidence metrics on domain-domain interactions, SPRY containing proteins are themselves often comprised of regions of high structural confidence (e.g. many available PDBs for RINGs, coils and SPRYs) but their relative arrangement within the molecule is unpredictable. Do isolated SPRYs show any better/worse binding to targets?

This is a good question and something we have been trying to address. We compared interaction profiles obtained using the full-length SPRY proteins with those obtained using the isolated SPRY domains. As shown in **Fig. 2a**, the SPRY domain accounts for the majority of contacting residues in high-scoring complexes, supporting the idea that it provides the principal substrate-recognition interface. However, **Fig. 2b-c** show that overall SPRY sequence similarity is only weakly associated with similarity in target profiles, suggesting that recognition cannot be solely explained by global SPRY-domain homology. When we directly compared the SPRY-only and full-length screens, we found moderate overall concordance (**Supplementary Fig. 2a**), and many of the high-confidence interactions highlighted in the manuscript were recovered in both datasets (**Supplementary Fig. 2b**). At the same time, some interactions differed between the two analyses. For example, in the hnRNPUL2:cGAS model, residues outside the SPRY domain contribute to the predicted interface, and the interaction is lost when the SPRY domain is modeled in isolation (**Supplementary Fig. 2c-d**). Conversely, for the closely related TRIM16 and TRIM16L proteins, the isolated SPRY domains show more similar binding profiles than the corresponding full-length proteins (**Supplementary Fig. 2e**). Thus, isolated SPRYs do not show uniformly stronger or weaker binding than full-length proteins; rather, these analyses indicate that the SPRY domain captures an important

component of substrate recognition, while the full-length protein context can either enhance or restrict.

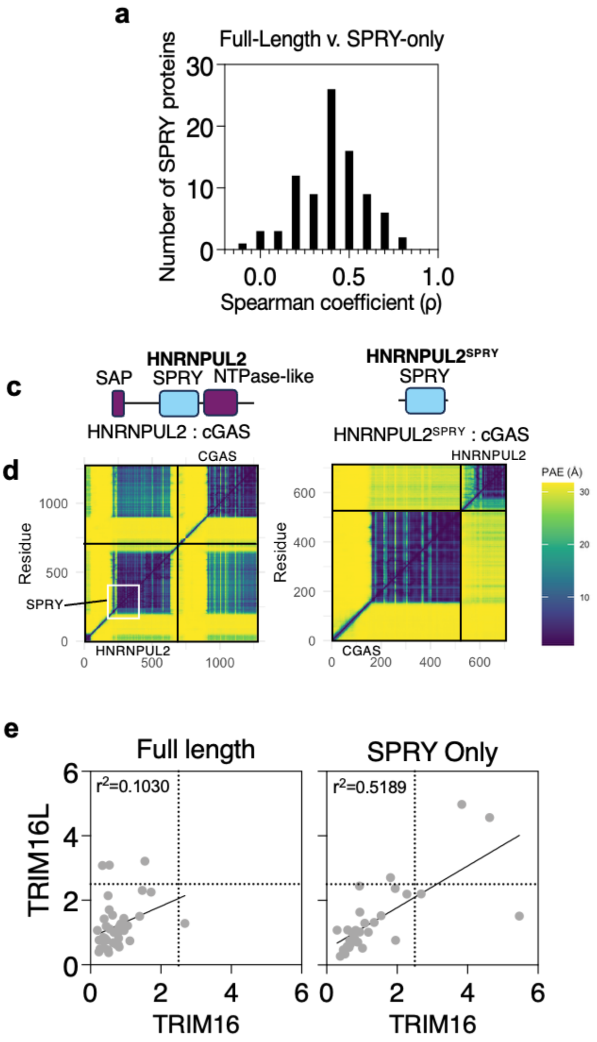

13. Technically, fig. 1f does not show that TRIM58 destabilises OAS1, as there is no condition with OAS1 alone. Perhaps alter the text to reflect this or repeat with the necessary control. The direction of the text is fine, as Fig. 1g provides a striking result, but 1f needs attention.

The reviewer raises an important consideration. To address this, we repeated the experiment using a OAS1 alone condition, as suggested. We found that OAS1 expression is substantially reduced when wildtype TRIM58 is co-expressed, but not when transfecting a catalytically inactive mutant of TRIM58 (C57S C60S) (**FigS1e**). This data better supports the conclusion that TRIM58 targets OAS1 for degradation in a manner that is dependent on its ubiquitin-ligase activity. We have included this new data to Supplementary Figure 1.

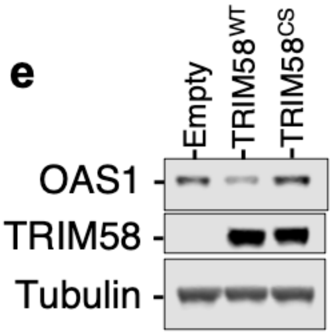

14. Fig. 2c - for clarity, please specify the meaning of the connecting lines between the bait 'hits' in the legend. What does the correlation coefficient relate to exactly? % similarity, is this across the whole molecule, or the PRYSPRY (presumably the latter would be a more useful comparison). And it is well established that single variations in SPRY variable loops can toggle binding, so this could be better referenced in the text. It would also be helpful to see e.g. dissimilar PRYSPRYs binding a common target, as PAE plots in the supplementary. Do any shared motifs occur at the variable loops between dissimilar SPRY molecules?

We agree that this figure needs more clarity. The connecting lines in Fig. 2c indicate protein-protein predictions with common sensors, i.e. connecting lines between the interaction score of ASH2L-MDA5 and the interaction score of TRIM51-MDA5. We only use % similarity of the SPRY domain alone, not the whole molecule. We have modified the figure legend to clarify this point and we include the PAE maps as supplementary information, as requested. We have searched for motifs in the variables loops but we did not find any similar patterns between dissimilar SPRY molecules.

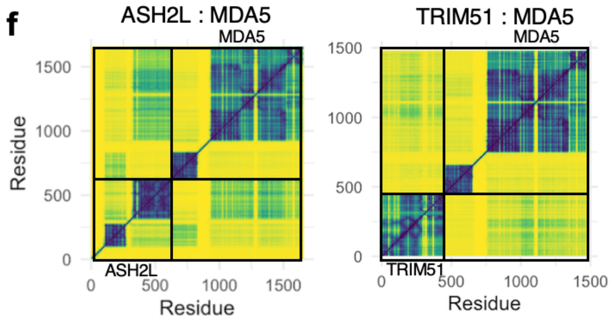

15. Fig. 2i - Bat RIG-I binds both TRIM25 and Riplet? This is in contrast to the predicted directionality in 2h?

The reviewer astutely noted that, in Fig.2i, pulling down bat RIG-I co-immunoprecipitated with both bat Riplet and bat TRIM25, while AlphaFold predictions only suggest a Riplet-RIG-I interaction. However, while bat Riplet and bat TRIM25 express at comparable levels in the input sample, bat Riplet was far more

enriched in RIG-I pulldowns than bat TRIM25. Our interpretation of this data is that, indeed, the interaction between bat Riplet-RIG-I is stronger than the interaction between bat TRIM25-Rig-I.

16. Fig. 3a-b, Sup Fig. 3c,d - IFNB1 transcript normalised to 3p-hRNA transfection in control NTC cells - the presentation chosen obscures the baseline IFNB1 levels in the different siRNA transfections. What is the fold induction of IFNB1 in the different cell lines?

We have included the fold induction for each cell line in the Non-Targeting Control (NTC) siRNA transfection using HeLa and A549 cells. All other siRNA transfections were normalized to the NTC. *IFNB1* fold induction is ~44-fold in HeLa and ~300-fold in A549 cells. *IFNL2* fold induction in A549 cells is ~1600-fold, while it was not detected under mock conditions in HeLa cells but detected in 3phRNA-transfected HeLa cells. We included this data in Supplementary Figure 4.

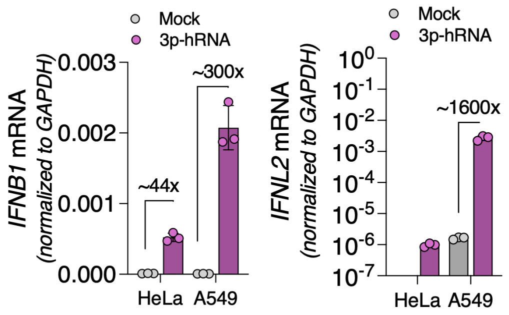

17. Fig. 3g - RLUs of EV-A71 is only tested in TRIM25 KO cells transfected with the Riplet T25 chimera. The full panel of cDNAs (parental E3s and the inverse 25-riplet swap) should be tested in parallel to confirm the effect is specific to TRIM25 PRYSPRY.

This is a great suggestion that will help clarify the role of different domains of TRIM25 in its antiviral activity. To answer this question, we tried to generate a TRIM25$^{-/-}$ Riplet$^{-/-}$ double knockout cell line but we couldn't get a stable clone. Instead, we generated a Riplet$^{-/-}$ A549 cell line (**FigZa**) in combination with a single siRNA to deplete TRIM25, and reconstituted these cells with CRISPR-resistant Riplet or chimeras and siRNA-resistant TRIM25 (**FigZb**). Of all reconstituted proteins, Riplet-T25^{SPRY} expressed at very low levels in this cell line, while the other proteins expressed at similar levels. Stimulating these cells with the RIG-I agonist 3phRNA showed that (1) ablation of Riplet expression completely abolished *IFNB* induction; (2) *IFNB* production is rescued when cells were reconstituted with a CRISPR-resistant Riplet but not TRIM25, (3) expression of TRIM25-Riplet^{SPRY} chimera can rescue *IFNB* production (**FigZc**). In terms of EV-A71 infection, reconstituting these cells with an siRNA-resistant TRIM25 substantially reduced virus infection, while T25-Riplet^{SPRY} had little impact virus replication (**FigZd**). Since the expression of Riplet-T25^{SPRY} in this cell line is much lower than the other chimeras, it is difficult to

draw conclusions about its impact of *IFNB* induction and virus replication. Therefore, in the manuscript, we included experiments using only T25-Riplet^{SPRY}.

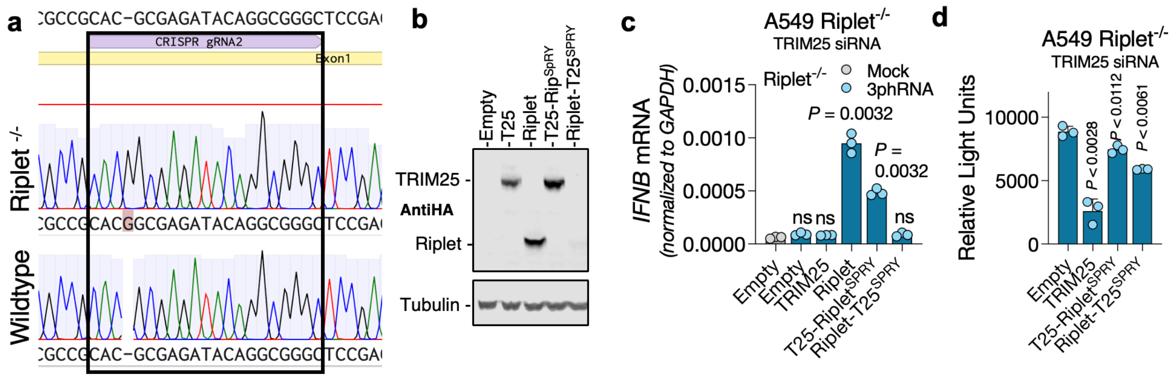

18. Fig. 4b - time point of 3p-hRNA transfection? Y-axis label suggested normalisation to NTC - incorrect label? What is the effect of bortezomib on IFNB1 mRNA in mock treated cells?

We thank the reviewer for spotting this typo, we have known corrected the axis label. We harvested cellular mRNA 8h post-transfection. Bortezomib-treatment slightly reduced the background expression of IFNB1 mRNA, but this signal is very close to the detection limit that it is difficult to draw conclusions. Nevertheless, the addition of bortezomib did not increase IFNB1 mRNA expression in the absence of a stimulus.

19. Fig. 4g - these experiments would benefit from an untransfected control cell to clarify how cDNA expression impacts sensor stability.

We agree with the reviewer and we have included an untransfected control.

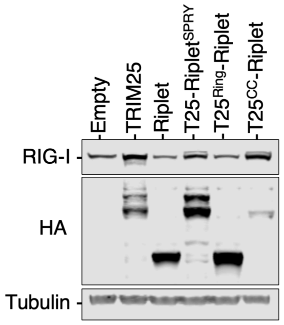

20. There seems to be an inverse correlation between sensor degradation and signaling output - is that the summary of Fig. 4? On the one hand, sensor degradation attenuates functional output (Fig. 4b), and the E3 that degrades the sensor is required for sensor function; on the other hand, changing coil-length in the E3 disables sensor degradation (Fig. 4g) but and enhances signaling response (Fig. 3j). Do the chimeras of panel Fig. g, h influence IFNB1 expression in the assay from Fig. 3j - this experiment would marry RIG-I expression with signal output.

We thank the reviewer for this insightful comment. We agree that an important question is whether Riplet-dependent effects on RIG-I abundance are mechanistically linked to Riplet-dependent signalling output.

First, the reviewer's reference to the former Fig. 4b is no longer applicable, as these inhibitor data were removed from the revised manuscript. In addition, our subsequent analysis did not support a simple interpretation that proteasome inhibition uniformly enhances signalling: whereas bortezomib increased IFNB1 induction, MG132 reduced it. We therefore concluded that global proteasome inhibition has pleiotropic effects in this assay and does not provide a reliable basis to infer a direct relationship between RIG-I turnover and signalling output.

We did, however, performed the reviewer's suggested experiment testing the ability of different chimeras to support RIG-I activation and subsequent IFN production. We used our Riplet$^{-/-}$ cells reconstituted with the different chimeras in combination with siRNA-mediated depletion of TRIM25. These experiments argue against a simple inverse correlation between RIG-I degradation and signalling. In particular, the construct in which the Riplet coiled-coil was replaced by the TRIM25 coiled-coil no longer reduced steady-state RIG-I abundance, yet it retained IFNB1 induction compared with WT Riplet. Conversely, the chimera bearing the TRIM25 RING in the Riplet backbone still reduced RIG-I abundance and remained signalling-competent. Together, these results indicate that Riplet-dependent control of RIG-I protein abundance and Riplet-dependent activation of IFNB1 are genetically separable outputs with distinct domain requirements. A caveat of these experiments is the relative low expression of the T25CC-Riplet chimera, which may influence the magnitude of IFNB fold induction.

We have revised the text to clarify this point and now avoid implying that RIG-I destabilisation is required for signalling. Rather, our data support a model in which the Riplet coiled-coil contributes to the effect on RIG-I stability, while robust signalling can be maintained in its absence.

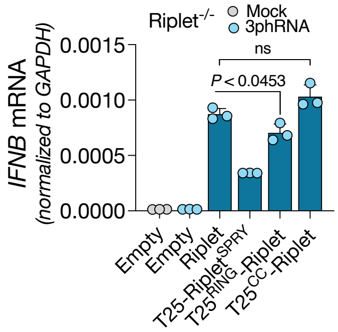

21. The data is generally clear. To facilitate their interpretation and for clarity, Western blots require size markers and Co-IPs should indicate the flag-/ha-epitope tags. Would make fig. 2 i-j much clearer, particularly given apparent co-migration of IgG (heavy chain?) and riplet, and the lack of control IPs.

We agree that contextual markings will improve the interpretation of these results. We added size markers to the western blots in fig2 in order to improve clarity.

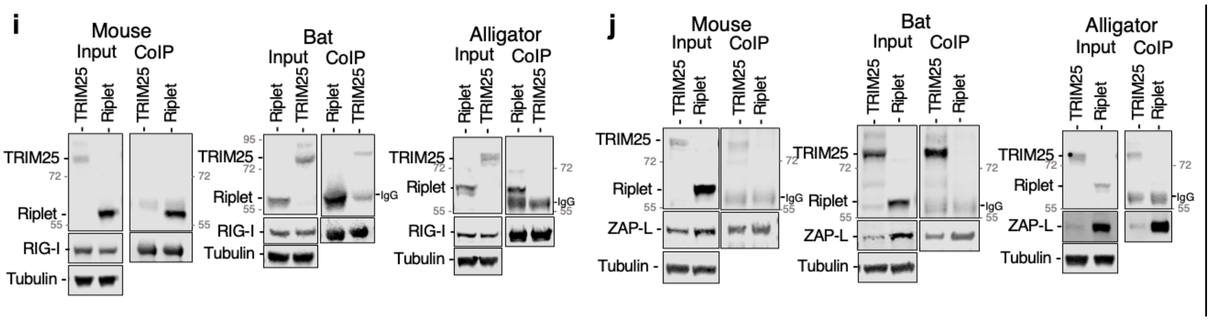

22. The figure legends could provide more detail.
We added additional experimental details (such as time points) to the figure legends.

Reviewer #2 (Significance (Required)):

The paper provides a rich resource of potential E3-sensor interactions and represents a conceptual and technical advance for the field. The authors take a novel approach to identify these pairings. Several pairings are validated in CoIPs, and two pairings (T25-ZAP, RIPLET-RIG-I) are studied in detail. Many E3s - including the TRIM proteins which comprise the bulk of E3s studied here - are purported to regulate key nucleic acid sensors in the literature, but the large scale approach taken here provides evidence that the pairings are really quite specific. The findings also supports prior work showing that the PRYSPRY domain (here called the SPRY) is a functionally plastic module that through variable loops can bind a range of different protein substrates.

The paper will be most interesting to the innate immune field, those working on nucleic acid sensing, and those looking at innate responses to RNA vaccines.

Regulation of E3 ubiquitin ligases, viral RNA sensing
